# Registration of coronary MRA to DCE-MRI myocardial perfusion series improves diagnostic accuracy through the computation of patient-specific coronary supply territories: a CE-MARC sub-study

**DOI:** 10.1186/1532-429X-16-S1-O25

**Published:** 2014-01-16

**Authors:** Constantine Zakkaroff, Aleksandra Radjenovic, John D Biglands, Sven Plein, John P Greenwood, Derek R Magee

**Affiliations:** 1 Division of Medical Physics, University of Leeds, Leeds, UK; 2 School of Computing, University of Leeds, Leeds, UK; 3 MCRC & LIGHT, University of Leeds, Leeds, UK; 4 Institute of Cardiovascular and Medical Sciences, BHF Glasgow Cardiovascular Centre, University of Glasgow, Glasgow, UK

## Background

It is generally acknowledged that the 17-segment AHA model provides a suitable approximation for mapping the results of X-ray angiography onto myocardial anatomy in a consistent way in the absence of a more exact method. In practice, coronary anatomy varies from patient to patient which is acknowledged as the main limitation of the AHA model. The aim of this study was to establish whether the generation of a patient-specific coronary artery to perfusion segment map improved diagnosis of myocardial ischaemia.

## Methods

In this retrospective sub-study with the data from the CE-MARC trial (Greenwood et al., Lancet, 2012) an 18-patient sample was selected where the image quality of the magnetic resonance angiography (MRA) data allowed for a reliable manual coronary tree annotation. Mediated spatiotemporal registration was used to compute the deformation fields between the MRA and perfusion data. The deformation fields were applied to the annotated coronary trees in order to warp them into the spatiotemporal coordinate space of the corresponding perfusion series. Three patient-specific coronary supply territories (RCA, LAD, LCX) were computed on the basis of the physical proximity of the major coronary arteries to the epicardial surface of the basal, medial and apical slices in the given perfusion series. Quantitative perfusion analysis using Fermi-constrained deconvolution was performed after manual motion correction and contour delineation in the perfusion series. Myocardial perfusion reserve (MPR) ratios were calculated from the ratio of stress to rest myocardial blood flow (MBF) estimates. A comparison between the diagnoses made using the AHA map and the patient-specific map was performed for each of the 18 patients. The presence of myocardial ischaemia was assessed using the consensus diagnosis of invasive, quantitative X-ray angiography and myocardial Single Photon Computed Tomography (SPECT) imaging.

## Results

**Figure 1:**
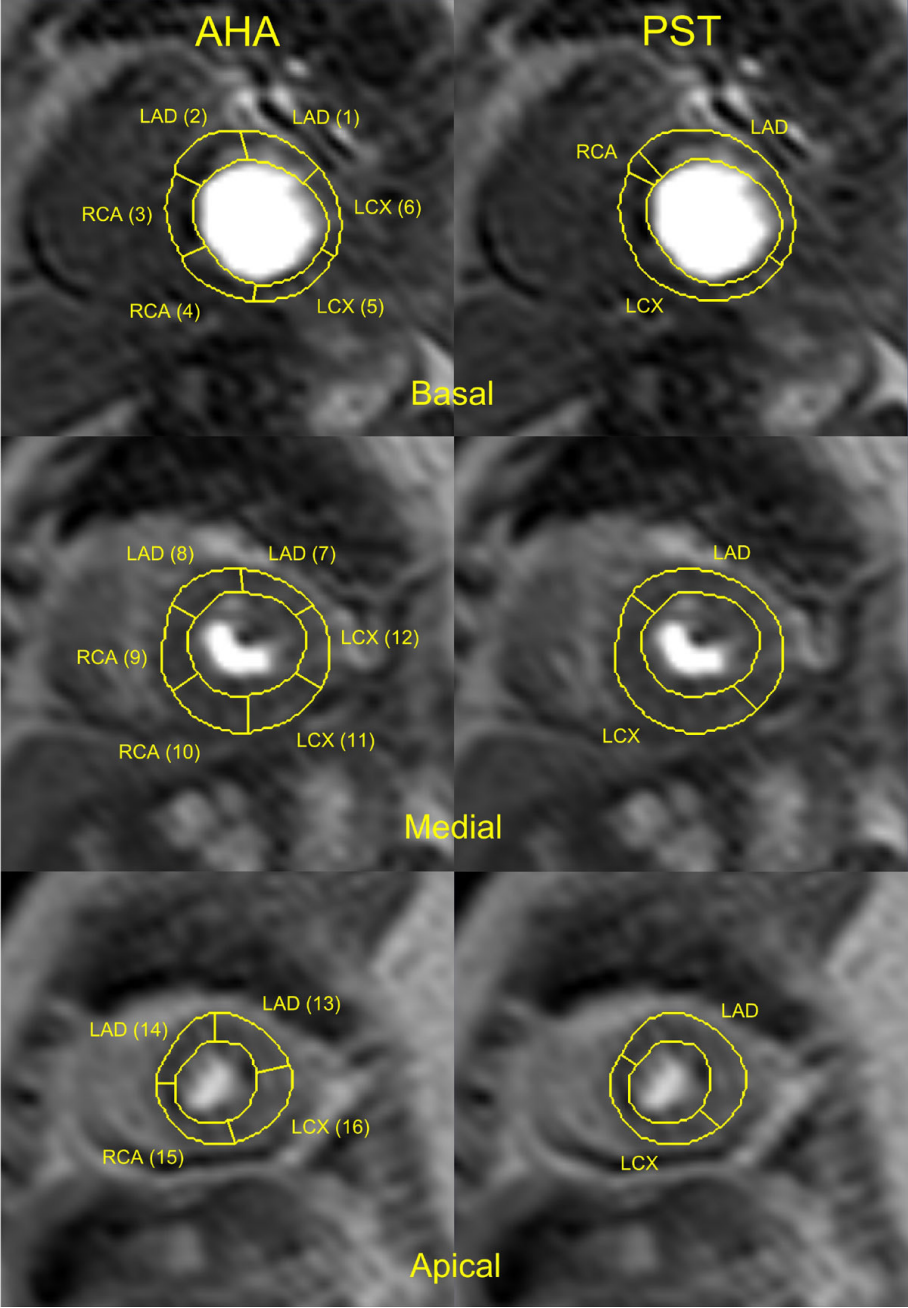
**A visual comparison between AHA and patient-specific coronary supply maps for a patient with a left-dominant coronary supply system**.

The patient-specific territories were markedly different from the AHA (Figure 1). For a total of 54 coronary arteries eight cases (15%) were diagnosed incorrectly with the AHA model, while only five cases (9%) were diagnosed incorrectly with perfusion analysis based on patient-specific coronary supply territories (Table 1). The results of perfusion quantification with patient-specific coronary territories for the RCA were less reliable than for the LAD and LCX artery.

**Table 1:** Fraction of coronary arteries correctly diagnosed with ischaemia with the AHA model and patient-specific territories (PST).

RCA		LAD		LCX	
AHA	PST	AHA	PST	AHA	PST
16/18 (89%)	14/18 (78%)	14/18 (78%)	17/18 (94%)	16/18 (89%)	17/18 (94%)

## Conclusions

The use of patient-specific blood supply territories derived from the actual coronary anatomy has the potential of enhancing the diagnostic accuracy of quantitative perfusion analysis by providing a mechanism for computing perfusion parameters for the artery-specific regions of the myocardium. An improvement on the allocation of coronary artery to myocardial region over the AHA recommendation has been shown.

## Funding

This work was funded by the Top Achiever Doctoral scholarship awarded by the Tertiary Education Commission of New Zealand.

